# Autoantibodies to beta-adrenergic and muscarinic cholinergic receptors in Myalgic Encephalomyelitis (ME) patients – A validation study in plasma and cerebrospinal fluid from two Swedish cohorts

**DOI:** 10.1016/j.bbih.2020.100107

**Published:** 2020-07-18

**Authors:** Annie Bynke, Per Julin, Carl-Gerhard Gottfries, Harald Heidecke, Carmen Scheibenbogen, Jonas Bergquist

**Affiliations:** a Analytical Chemistry and Neurochemistry, Department of Chemistry – BMC, Box 599, Uppsala University, 75124, Uppsala, Sweden; b The ME/CFS Collaborative Research Centre at Uppsala University, Sweden; c Department of Neurobiology, Care Sciences and Society, Karolinska Institutet, Stockholm, Sweden; d ME/CFS-policlinic, Neurological Rehabiliation Clinic, Stora Sköndal, Stockholm, Sweden; e Gottfries Clinic, Affiliated with Institute of Neuroscience and Physiology, The Sahlgrenska Academy, Göteborg University, Sweden; f CellTrend GmbH, Luckenwalde, Germany; g Institute for Medical Immunology, Charité-Universitätsmedizin Berlin, Berlin, Germany

**Keywords:** Myalgic encephalomyelitis (ME), Autoantibodies, Beta-adrenergic receptors, Muscarinic cholinergic receptors, Plasma, Cerebrospinal fluid

## Abstract

Myalgic encephalomyelitis (ME) also known as ME/CFS (Chronic Fatigue Syndrome) or ME/SEID (Systemic Exertion Intolerance Disorder), is a disabling and often long-lasting disease that can drastically impair quality of life and physical/social functioning of the patients. Underlying pathological mechanisms are to a large extent unknown, but the presence of autoantibodies, cytokine pattern deviations and the presentation of cognitive and autonomic nervous system related symptoms provide evidence for ME being an immunological disorder with elements of autoimmunity. Increased levels of autoantibodies binding to adrenergic and muscarinic receptors in ME-patients have been reported. It is hypothesized that these autoantibodies have pathological significance and contribute to the ME-specific symptoms, however, these observations need to be validated.

This study was designed to investigate potential differences in adrenergic and muscarinic receptor autoantibody levels in plasma and cerebrospinal fluid (CSF) samples between ME patients and gender and age-matched healthy controls, and to correlate the autoantibody levels to disease severity.

We collected bodyfluids and health-related questionnaires from two Swedish ME cohorts, plasma and CSF from one of the cohorts (n ​= ​24), only plasma from the second cohort (n ​= ​24) together with plasma samples (n ​= ​24) and CSF (n ​= ​6) from healthy controls.

All samples were analysed for IgG autoantibodies directed against Alpha- (α1, α2) and Beta- (β1-3) adrenergic receptors and Muscarinic (M) 1–5 acetylcholine receptors using an ELISA technique. The questionnaires were used as measures of disease severity.

Significant increases in autoantibody levels in ME patients compared to controls were found for M3 and M4 -receptors in both cohorts and β1, β2, M3 and M4-receptors in one cohort. No significant correlations were found between autoantibody levels and disease severity. No significant levels of autoantibodies were detected in the CSF samples. These findings support previous findings that there exists a general pattern of increased antibody levels to adrenergic and muscarinic receptors within the ME patient group. However, the role of increased adrenergic and muscarinic receptor autoantibodies in the pathogenesis of ME is still uncertain and further research is needed to evaluate the clinical significance of these findings.

## Highlights

• Myalgic Encephalomyelitis (ME) is a devastating disorder (with millions of patients worldwide) with unclear aetiology and no clear diagnostic biomarker available.
• This study support the existance of a general pattern of increased antibody levels to adrenergic and muscarinic receptors within the ME patient group.
• No evidence for intrathecal antibody production was found in cerebrospinal fluid. The role of increased autoantibodies in the pathogenesis of ME is still uncertain and further research is needed to evaluate their clinical significance.

## 1 Introduction

Myalgic encephalomyelitis (ME) means “muscle pain related to central nervous system inflammation” and was described in the 1950’s (Acheson, 1957; Galpine and Brady, 1957) as a complex, often severely disabling and chronic illness with unknown aetiology and pathological mechanism. Worldwide, millions of people are affected and prevalence is estimated to 0.2–0.4% with a peak within women 10–19 years and 30–39 years (Mensah et al., 2017; Nacul et al., 2011; Brurberg et al., 2014). Despite the seriousness of the illness, no specific diagnostic biomarkers or disease modifying treatments are available. The diagnosis is based on clinical criteria, such as the Canadian consensus criteria (CCC, 2003) (Carruthers et al., 2003) and the International consensus criteria (ICC, 2011) (Carruthers et al., 2011) or the more recent Institute of Medicine (IOM) criteria (2015) (Institute of Medicine, 2015).

Post-exertional malaise (PEM) is central to the symptomatology and a required symptom for diagnosis in current diagnostic criteria, it is the significant worsening of disease symptoms upon physical or mental exertion. Other common symptoms include pain, headache, vertigo (orthostatic), unrefreshing sleep, muscle weakness, gastrointestinal problems, newly acquired drug/food allergies, recurrent throat pain, light sensitivity, flu like symptoms and several neurocognitive symptoms such as working memory impairment, concentration and information processing difficulties.

ME is also known as chronic fatigue syndrome (CFS), however, there is some discrepancy to the clinical definition between ME and CFS. For example, the CFS criteria presented by Fukuda et al., 1994) (Fukuda et al., 1994) do not require PEM, which is central to the ME definition. The US center for disease control (CDC) now recommends the IOM criteria for diagnosis (https://www.cdc.gov/me-cf, 2019) since the Fukuda definition largly overlaps with recent criteria for stress related exhaustion disorder (Grossi et al., 2015; Wallensten et al., 2016). Therefore, we also decided to use the term ME as it more clearly defines a patient group with PEM as core symptom using CCC, ICC or IOM criteria for diagnosis.

The combination of symptoms referred to as symptom clusters, illness severity, co-morbidities, disease trigger and treatment response vary widely between the patients, which has led to the belief that there exist several clinical subgroups within the patient group (Nagy-Szakal et al., 2017; Jonsjo et al., 2017). Thus far, no consensus about aetiology or pathology exists and arrays of alternative hypotheses are continually being tested. Among the most established pathological theories are; metabolic-, immune system- and neuroimune-dysfunctions together with existence of autoimmunity. Evidence for the role of autoimmunity in ME include the detection of autoantibodies directed against several different proteins such as; Cardiolipin, Anti-nuclear antibodies Thyroid peroxidase, adrenergic and muscarinic receptors and oxidative/nitrosative caused antigens. Many of these autoantibodies are involved in the pathogenesis of other diseases, making it possible that this is the case also in ME. However, much of the evidence for autoimmunity is indirect and circumstantial and greater proof and understanding of the pathological processes linked to autoimmunity is needed (Underhill, 2015; Blomberg et al., 2018; Franziska Sotzny et al., 2018; Steven et al., 2011).

Scheibenbogen et al. (Loebel et al., 2016) recently made an attempt to provide evidence of autoimmunity as part of the pathological explanatory model for the chronic immune system activation, autonomous dysregulation and metabolic dysfunction seen in ME. By screening serum obtained from patients (n ​= ​268) and controls (n ​= ​108) for the presence of IgG autoantibodies against Alpha (α) and Beta (β) adrenergic (α1, α2, β1, β2, β3) receptors, Muscarinergic cholinergic receptors (M1-M5), dopamine receptors, serotonin receptors, angiotensin receptors and endothelin receptors, using a specific ELISA technique developed and provided by CellTrend. Results showed significantly higher autoantibody levels against β2, M3 and M4 receptors in ME-patients compared to controls, suggesting a possibility that deviant autoantibody levels might be a characteristic for ME but also that the autoantibodies might contribute to the symptomatology and disease severity.

Autoantibodies directed against different receptors have pathological relevance in several autoimmune diseases with a similar symptomatology as ME, these include Postural Orthostatic Tachycardia Syndrome (POTS), Chronic regional pain syndrome (Fluge et al., 2015), Sjogren’s syndrome, Hypothyroidism and Fibromyalgia (Franziska Sotzny et al., 2018). It has been suggested that adrenergic and muscarinic autoantibodies interfere with the binding of norepinephrine/epinephrine, influence the normal receptor function and contribute to symptoms seen in ME.

Adrenergic receptors are involved in the normal function of the autonomous nervous system by regulating the sympathetic and parasympathetic reactions that control energy metabolism, immune system activation, heart muscle activity and neurocognitive function. Muscarinic receptors are important for neurological and neuromuscular transmission. Dysfunction in adrenergic and muscarinic receptors introduced by autoantibody-binding have been suggested to contribute to autonomic dysfunction associated with symptoms of orthostatic intolerance, vertigo, bladder dysfunction, malaise, gastro intestinal disturbances, short term memory loss, concentration difficulties, muscle weakness and problems with information processing (Loebel et al., 2016; Johnston et al., 2016; Haga et al., 2012). However, little evidence exists regarding the receptor-autoantibody interactions and pathological significance of the autoantibodies leaving a knowledge gap that needs to be filled.

It is yet to be explored whether the autoantibodies to Beta-adrenergic and muscarinergic cholinergic receptors have any clinical significans, whether they bind to the receptors and whether treatment options including blood purificating techniques (eg plasmaphoresis or immunoadsorbtion) improve symptoms and/or disease severity (Scheibenbogen et al., 2018).

This study was designed to 1) replicate the previous findings of increased autoantibodies in 2 independent Swedish cohorts 2) analyse if elevated autoantibodies are present in CSF and 3) to examine the correlation between autoantibody levels and clinical symptoms.

Investigations were performed by screening plasma and CSF collected from Swedish ME-patients for presence of autoantibodies binding to adrenergic and muscarinic cholinergic receptors.

## 2 Material and methods

### 2.1 Human plasma and CSF samples

Study participants were diagnosed at the Stora Skondal clinic in Stockholm (SK) and at the Gottfries clinic in Göteborg (GC) between years 2013–2018. The SK sample was collected after approval from the regional ethic committee in Stockholm 2016/4:7 and the GC sample after approval from the regional committee in Göteborg 2016:966–15. All methods and sampling were carried out in accordance with relevant guidelines and regulations including the Declaration of Helsinki.

All patients at both Stora Skondal and Gottfries clinics fulfilled the Canadian Concensus Criteria, the International Concensus Criteria and the IOM criteria (Carruthers et al., 2003; Carruthers et al., 2011; Institute of Medicine, 2015). In total, 48 patients were included, 24 from each clinic respectively, making up two independent cohorts. Patient characteristics for both groups are presented in Table 1. 50% of the SK-patients and 33% of the GC-patients suffered from co-morbidities. The two most common co-morbidities in both groups were Hypothyriodosm and Fibromyalgia, but other diseases commonly seen in ME, such as Irritable Bowel Syndrome (IBS) were also present (Franziska Sotzny et al., 2018). Information about disease triggers were only obtained for the SK-patients where infection was the trigger of ME in 87.5% of the cases. The two most common disease-triggering infections were the Flu and Upper respiratory infection and other occurring disease-triggering infections were Neuroborreliosis, TWAR, Epstein Barr virus, Gastroenteritis and Meningitis.

**Table 1:** Patient characteristics.

Patient group	Age (years)	Gender	Co-morbidity	Disease duration prior sampling (years)	Disease onset	Infection triggered disease
SK- patients	20-56 (M:40, SD:11)	Female:16, Male:8	50%	2-25 (M:8.3, SD:6.4)	Fast: 21 Slow: 3	87.5%
GC- patients	18-63 (M:43, SD:12.2)	Female:16, Male:8	33%	4-27 (M:10.25, SD:7.3)	Fast: 8 Slow:16	No information obtained

Patient characteristics. Age and disease duration prior to sampling are presented as minimum value and maximum. Mean (M) and standard devations (SD) are shown within brackets.

Patient plasma samples were collected at a patient baseline assessment visit. 24 plasma samples were collected at each clinic and 24 cerebrospinal fluid samples were collected at the Gottfries clinic. Health related questionnaires were completed and handed in at the sampling occasion and a written consent was signed. In addition to the patient samples, plasma samples were collected for 24 age and gender-matched controls from the blood bank in Uppsala Academic Hospital together with 6 CSF samples from age and gender-matched controls from the biobank at Uppsala Clinical Research Centre. All samples were shipped to the CellTrend Laboratory in Lückenwalde, Germany as coded, randomised and blinded samples.

### 2.2 Health related questionnaires

In addition to the plasma and CSF samples, several health related questionnaires were collected from the two clinics, six from the SK-clinic and three from the GC-clinic. Questionnaires collected from the SK-clinic included; RAND-36, EQ-5D, Hospital anxiety and depression scale (HADS), ME/CFS symptom related formula (ME/CFS-SRQ) (Blomberg et al., 2018) and Fatigue Severity Scale (FSS) (Franziska Sotzny et al., 2018). Collected questionnaires from the GC-clinic included Fibro Fatigue Scale (FFS), Becks Depression Inventory (BDI) and Mental Fatigue Scale (MFS). The symptom formulas were collected for two main purposes. The first purpose was to yield a deeper understanding of physical and mental well being, self-experienced quality of life and disease profile/symptomatology among the patients. The second purpose of the formula collection was to enable correlation analyses between patient questionnaire scores and levels of receptor autoantibodies, trying to find possible associations between increased/deviant autoantibody levels and symptomatology/disease severity. Appendix 1 provide a brief description of the health related questionnaires used in this study.

### 2.3 Autoantibody quantification- ELISA

**Fig. 1:**
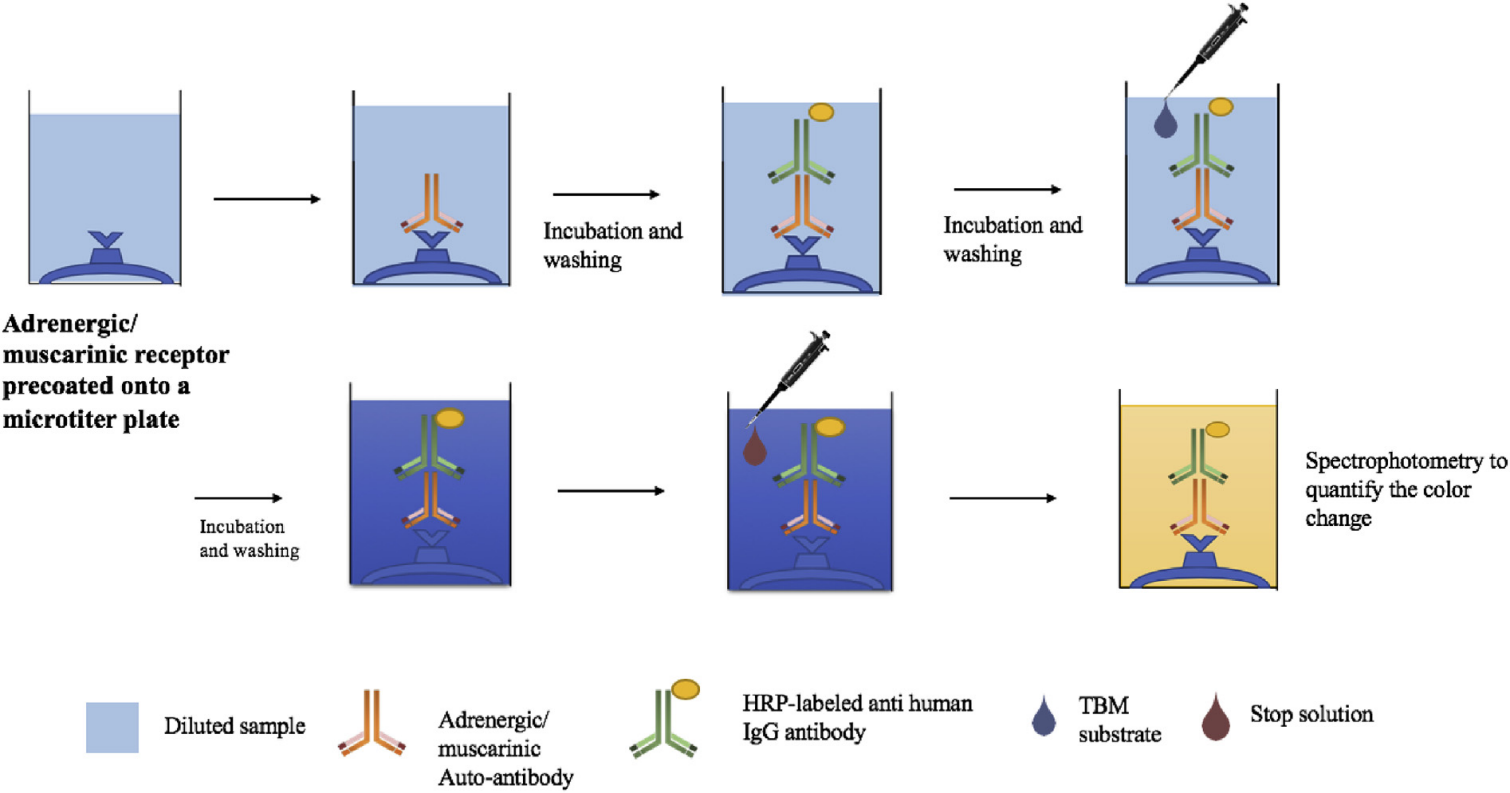
Method visualization of the CellTrend ELISA technique used to quantify sample autoantibody levels.

All collected samples were shipped to the CellTrend laboratory in Luckenwalde, Germany where immunoassays were performed to identify presence of autoantibodies to Alpha and Beta-adrenergic receptors and muscarinic cholinergic receptors (Fig. 1). GC-samples were collected earlier in time than the SK-samples and where therefore shipped and analysed separately. The GC-samples were tested for β1, β2, β3, M3 and M4 in plasma and cerebrospinal fluid while the SK- samples were tested for all of the 10 receptor types (α1, α2, β1, β2, β3, M1, M2, M3, M4 and M5) in plasma.

All antibodies were quantified by ELISA (CE-certified for anti-β_1_AR Ab, anti-β_2_AR Ab, anti-M3 Ab and M4 Ab). As described previously (Cabral-Marques et al., 2018), microtiter 96-well polystyrene plates were coated with extracts from transfected Chinese Hamster Ovary cells overexpressing the human GPCRs. Conformational epitopes of the receptor were maintained by adding 1 ​mM calcium chloride to each buffer. Duplicate samples of a 1:100 dilution were incubated at 4–8 ​°C for 2 ​h. After washing, plates were incubated for 60 ​min with a 1:20,000 dilution of horseradish peroxidase-labelled goat anti-human IgG (Jackson, Bar Harbor, ME, USA) for detection. All samples were coded and analysed for GPCR Abs assessment by individuals who had no information regarding the patients and controls characteristics and distribution. After three rounds of washing with 300 ​μL wash buffer 100 ​μL TBM substrate was dispensed into each well to allow for a reaction between HRP and Tetramethylbenzidine (TBM) creating a change of colour of the solution to blue. Before the stop solution was added a third round of incubation during 20 ​min at room temperature in a dark room was performed. As a final step 100 ​μL stop solution (sulphuric acid) was added to the wells and a change in colour to yellow with a maximal absorbance at 450/620 ​nm was obtained. The colour intensity is proportional to the amount of HRP activity, which in turn is related to the levels of target analyte. Spectrophotometry was used to quantify the colour change, which is proportional to the amount of autoantibodies within the sample.

### 2.4 Statistical analysis

All statistical analyses were done using R studio 1.1.4 and were supervised by an experienced statisticians. All included data were transformed to logarithmic prior to analyses.

#### 2.4.1 Hypothesis testing

A Sharpio-Wilk test was used to test the normality of distribution within the population and revealed that data for most of the receptor autoantibodies were unequally distributed, therefore, a Wilcoxon signed rank test was used for hypothesis testing.

Hypothesis testing determined whether there were any significant differences in levels of receptor-antibodies between patients and controls. Three separate comparisons were made, two comparing plasma-antibody levels in controls compared to SK- and GC-patients separately and one comparing CSF-autoantibody levels in GC-group compared to controls. Following hypothesis testing, Bonferroni correction was carried out to decrease the risk of type 1 errors. New significance levels were set to 0.005 for the SK-group and 0.01 for the GC-group.

In addition to the hypothesis testing, the percentage of sampled individuals with increased antibody levels to at least one of the receptor types was evaluated. Increased autoantibody levels were defined as values above the 90th percentile of the control group. These calculations were done with the purposes of investigating the general pattern of antibody level deviations within the two patient cohorts and to enable comparisons with former study results, showing elevated antibody levels in one third of the sampled patients (Loebel et al., 2016).

#### 2.4.2 Principal component analyses

Principal component analysis (PCA) was used to prepare the data for multivariate analysis and to discover important features, differences and relationships within the patient groups and between patients and controls. Deviant antibody levels were considered levels outside of the 95% confidence interval of controls.

#### 2.4.3 Correlation analyses, antibody levels and disease severity

Correlation analyses between patient questionnaire scores and receptor-autoantibody levels were made using the Spearman Rank Correlation Test. The purpose of the analyses was to investigate possible correlations between receptor antibody levels and disease severity.

Individual formula scores on all of the collected questionnaires were used as a disease severity outcome measure and were correlated to adrenergic and muscarinic receptor antibody levels (units/mL). The Spearman method was chosen prior to the Pearson method based on the characteristics of the data. Significance level was set to p ​= ​0.001.

## 3 Results

### 3.1 Autoantibody levels, patients compared to controls

**Fig. 2:**
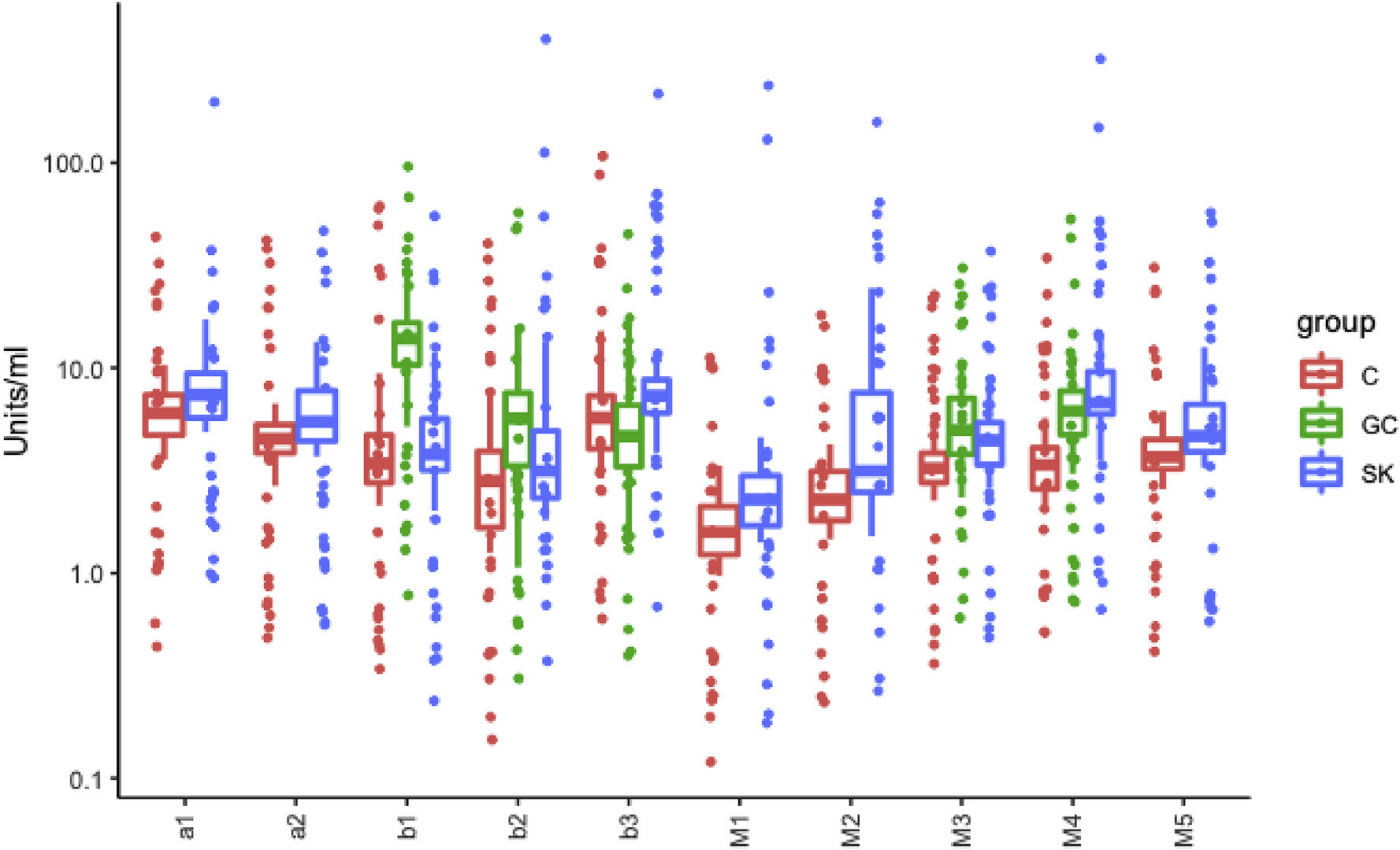
**Plasma-autoantibody levels, patients compared to controls** Boxplot visualizing results from Wilcoxon signed rank test. Blue boxes, Stora Skondal (SK) patients, green boxes Gottfries Clinic (GC) patients and red boxes healthy controls. Significant differences of autoantibody-levels between SK- and GC-patients and controls are seen for M3 and M4. Significant differences in autoantibody levels between GC-patients and controls are seen for *β*1, *β*2, M3 and M4. (For interpretation of the references to colour in this figure legend, the reader is referred to the Web version of this article.)

We found significantly increased autoantibody levels for M3 and M4-receptors within the SK-patients compared to controls and significantly increased autoantibody levels for β1, β2, M3 and M4 receptors in GC-patients compared to controls. Results are presented in Table 2 and visualized by boxplots in Fig. 2. In total 25% of controls, 79% of SK-patients and 91% of GC patients had at least one increased receptor autoantibody.

**Table 2:** Wilcoxon test results of autoantibody level comparisons between patients and controls.

*Autoantibodies (Units/mL)*	Stora Skondal (SK)	Gottfries Clinic (GC)
α1-Adr-Receptor	0.0266	ND
α2-Adr-Receptor	0.0158	ND
β1-Adr-Receptor	0.5881	1.0521e-08∗∗∗∗
β2-Adr-Receptor	0.1939	2.0806e-04∗∗∗
β3-Adr-Receptor	0.0354	0.0677
M1-Musc-Receptor	0.0076	ND
M2-Musc-Receptor	0.0188	ND
M3-Musc-Receptor	0.0030∗	1.5682e-05∗∗∗∗
M4-Musc-Receptor	1.9048e-06∗∗∗∗	1.5682e-05∗∗∗∗
M5-Musc-Receptor	0.0055	ND

Table showing results from Wilcoxon signed rank test with Bonferroni corrected p-values of.

∗p ​≤ ​0.005,∗∗p ​≤ ​0.001, ∗∗∗p ​≤ ​0.0001, ∗∗∗∗p ​≤ ​0.00001. ND ​= ​not determined.

**Fig. 3:**
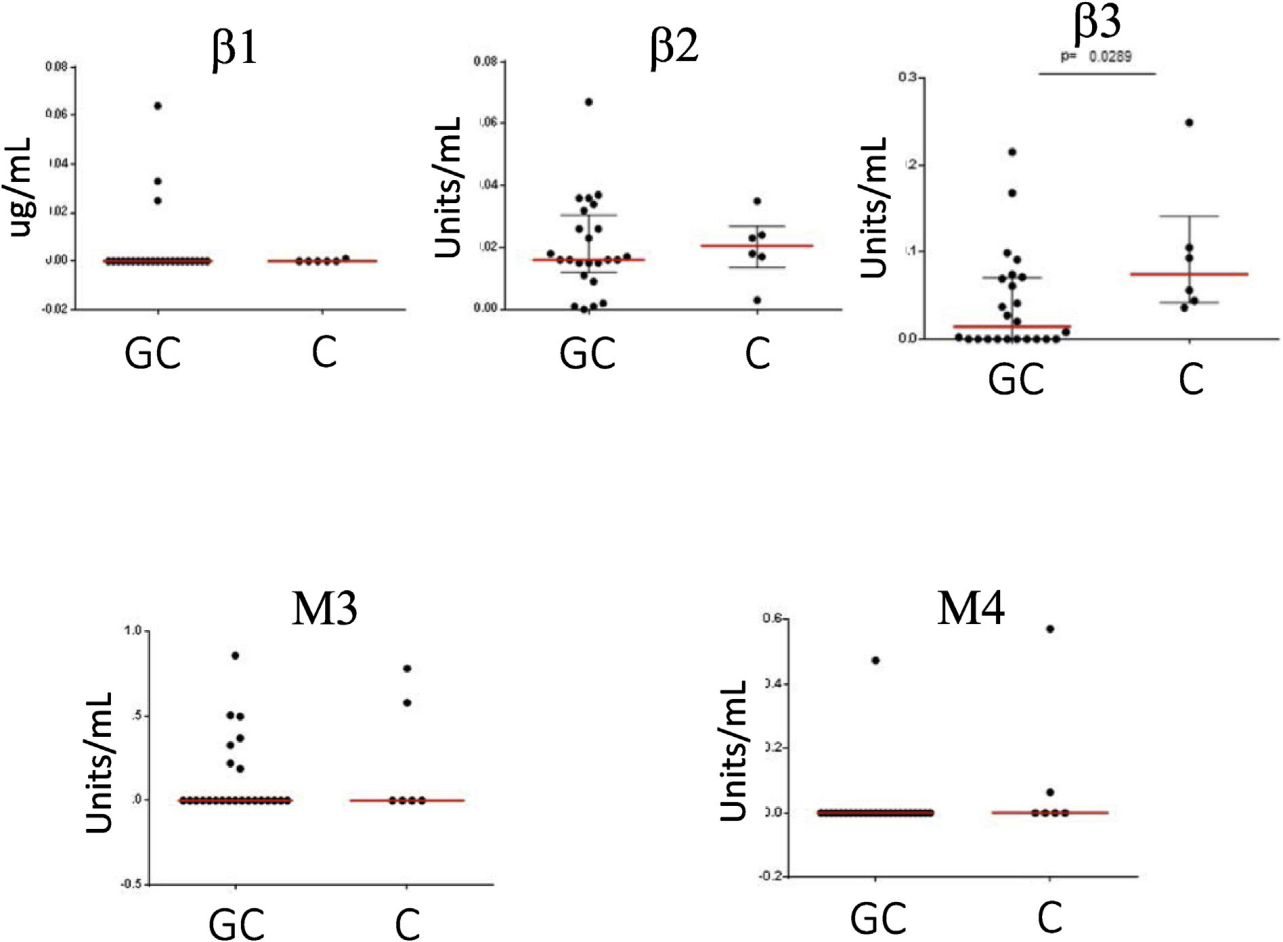
**Autoantibody levels in cerebrospinal fluid from GC patients compared to controls** Comparisons of *β*1-, *β*2-and *β*3-adrenergic and M3-, M4-receptor autoantibodies in cerebrospinal fkuid between ME/CFS patients from the Gottfries Clinic (GC) and healthy donor controls (C). Red lines represent the mean for each group and no significant differences existed between patients and controls except for the *β*3-adrenergic receptor were controls had slightly increased levels of detected autoantibody response, most likely related to background signal. (For interpretation of the references to colour in this figure legend, the reader is referred to the Web version of this article.)

GC-patients and controls had undetectable CSF-autoantibody levels within a noisy background and no significant differences in CSF-autoantibody levels (units/mL) between GC-patients and controls were found. Comparisons of CSF-autoantibody levels are visualized in Fig. 3.

**Fig. 4:**
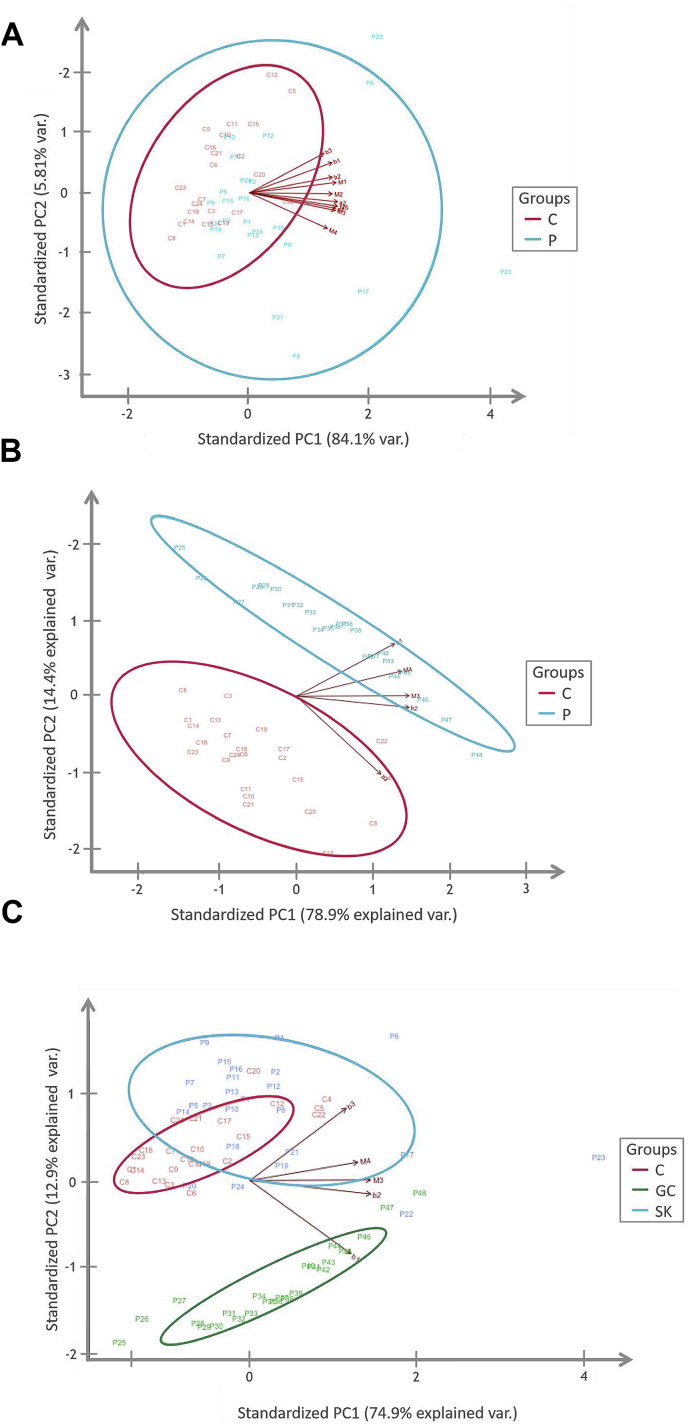
**a. PCA plot- SK-patients versus Controls** P ​= ​patients and C= Controls. Red colours represent the controls and blue colours the Stora Skondal (SK) patients. Eclipses present the 95% confidence intervals for each group. Patients outside of the red eclipse, the 95% C.I for controls were considered deviant and defined as group 1. **b. PCA-plot- GC- patients versus Controls** Red ​= ​controls, blue (P) ​= ​Gotttfries Clinic patients. Eclipses present the 95% confidence intervals for each group. Lines labelled as *β*1, *β*2, M3 and M4 represent autoantibody levels. Figure shows a distinct difference between GC-patients and controls. **c. PCA plot GC- patients, SK- patients and Controls** C ​= ​controls, P ​= ​patients. Red equals controls, blue the Stora Skondal patients and green the Gottfries Clinic patients. Eclipses represents the 95% confidence interval for each group. Clear differences in antibody patterns are apparent in the plot. SK–patients show more scattered values than both the controls and GC-patients and have a larger overlap with the controls. . (For interpretation of the references to colour in this figure legend, the reader is referred to the Web version of this article.)

Three separate PCA-analyses were conducted, one comparing SK-patients to controls, one comparing GC-patients to controls and one comparing both patient groups with the control group. PCA results are presented as plots in Fig. 4a–c. Ellipses represent the 95% confidence interval and were added to make distinctions between patients with and without deviant antibody patterns.

### 3.2 Correlation of autoantibody levels with symptomatology and disease severity

Spearman rank correlation tests were made to find possible correlations between patient questionnaire scores and antibody levels. Included questionnaires were; RAND physical component (RAND.PCS), RAND mental component (RAND.MCS), Fatigue severity scale (FSS), EQ-5D, EQ-5D-VAS, Hospital anxiety and depression scale, anxiety score (HAD.A), hospital anxiety and depression scale, depression score (HAD.D), ME/CFS-health related questionnaire summa score (S.Sum), ME/CFS-health related questionnaire PEM-score (S.PEM), Beck’s depression inventory (BDI) and Mental fatigue scale (MFS). Using corrected p-value of 0.001, due to multiple comparisons, no significant correlations were found between antibody levels and any questionnaire scores.

The health-related questionnaires were completed at the time of plasma-sampling and represent the patient’s physical and mental health, health related life quality and disease severity at this period. Questionnaire scores were used and interpreted to gain understanding about disease profiles and disease severity in the two patient cohorts and to enable correlation analyses. Individual formula scores are not presented, however patient mean and standard deviation are presented in Table 3, Table 4. Table 3 present scores on Hospital Anxiety and Depression Scale (HAD.A, HAD.D), ME/CFS Symptom Related Questionnaire (ME/CFS-SRQ), Fibro Fatigue Scale (FFS), Beck’s depression inventory (BDI) and Mental Fatigue Scale (MFS). Scores on RAND-36, EQ-5D and Fatigue Severity scale (FSS) are presented in a separate table, Table 4, as they were compared to population norm values for interpretation purposes. All the health-related questionnaires and their lack of correlation with individual levels of autoantibodies are presented in Table 5, and in the supplemental material Appendix.

**Table 3:** Mean and standard deviation (SD) on health related questionnaires; Hospital anxiety and depression scale, Anxiety score (HAD.A),; Hospital anxiety and depressions scale, depression score (HAD.D), ME/CFS symptom related questionnaire (ME/CFS-SRQ), ME/CFS symptom related questionnaire, Post exertional malaise score (ME/CFS –SRQ- PEM), Fibro fatigue scale (FFS), Beck’s depression inventory (BDI), Mental fatigue scale (MFS).

Questionnaire Scores	Mean	SD
HAD.A	4.91	3.69
HAD.D	7.26	3.58
ME/CFS -SRQ	29.95	9.92
ME/CFS –SRQ- PEM	5.56	2.48
FFS	33.84	7.24
BDI	16.46	9.05
MFS	22.81	6.30

**Table 4:** Patient mean, standard deviation (SD) and deviation from norm in absolute measurements, compared to normative data for RAND physical component (RAND.PCS), RAND mental component (RAND.MCS), RAND physical functioning (RAND.PF), EQ-5D Visual Analogue Scale (EQ-5D-VAS) and Fatigue Severity Scale (FSS).

Questionnaire	RAND.PCS	RAND.MCS	RAND.PF	EQ-5D-VAS	FSS
Patient mean values	22.60	44.12	38.89	31.8	6.61
SD	8.06	10.30	20.97	15.28	0.38
Mean norm values	51.36	50.63	90.85	80.7	2.3
SD	8.41	9.50	15.39	ND	0.7
Deviation from norm in absolute measurements	28.2	7.78	51.96	52.9	4.3

Patient mean scores on RAND.PCS, RAND PF, EQ-5D.VAS and FSS deviate notably from normative data, indicating severe fatigue, explicit physical limitations and decreased health related life quality among the patients. ND ​= ​not determined.

**Table 5:** Correlation analysis between the individual autoantibodies and the various questionnares.

Autoantibodies (Units/mL)	*RAND.PCS*	*RAND.MCS*	*FSS*	*EQ.5D*	*EQ.5D.VAS*	*HAD.A*
α1	e: 0.31 P: 0.89	e: 0.16 P: 0.46	e: 0.11 P: 0.59	e: −0.02 P: 0.93	e: −0.08 P: 0.70	e: 0.02 P: 0.93
α2	e: −0.21 P: 0.32	e: 0.33 P: 0.12	e: 0.16 P: 0.44	e: 0.15 P: 0.47	e: −0.07 P: 0.74	e: −0.10 P: 0.64
β1	e: −0.25 P: −0.25	e: 0.37 P: 0.08	e: 0.02 P: 0.91	e: 0.01 P: 0.97	e: 0.04 P: 0.85	e: −0.21 P: 0.33
β2	e: −0.19 P: 0.39	e: 0.35 P: 0.10	e: 0.13 P: 0.53	e: 0.11 P: 0.61	e: 0.16 P: 0.46	e: −0.07 P: 0.76
β3	e: 0.08 P: 0.73	e: 0.24 P: 0.26	e: 0.17 P: 0.43	e: −0.14 P: 0.51	e: 0.14 P: 0.52	e: −0.12 P: 0.58
Autoantibodies (Units/mL)	***HAD.D***	***S.Sum***	***S.PEM***	***BDI***	***MFS***	***FFS***
α1	e: −0.16 P: 0.47	e: 0.03 P:0.90	e: −0.07 P: 0.74	e: ND P: ND	e: ND P: ND	e: ND P: ND
α2	e: −0.15 P: 0.49	e:0,09 P: 0.69	e: 0.07 P: 0.75	e: ND P: ND	e: ND P: ND	e: ND P: ND
β1	e: −0.24 P: 0.28	e: 0.08 P: 0.72	e: 0.09 P: 0.67	e: 0.52 P: 0.07	e: 0.02 P: 0.95	e: −0,02 P: 0,47
β2	e: −0.07 P: 0.76	e:0.08 P:0.70	e: 0.19 P: 0.39	e: 0.53 P: 0.06	e: 0.03 P: 0.91	e: −0.18 P: 0.47
β3	e: −0.20 P: 0.35	e: 0.11 P:0.62	e: 0.02 P: 0.93	e: 0.53 P: 0.06	e: 0.03 P: 0.91	e: 0.18 P: 0.47
Autoantibodies (Units/mL)	*RAND.PCS*	*RAND.MCS*	*FSS*	*EQ.5D*	*EQ.5D.VAS*	*HAD.A*
M1	e: 0.14 P:0.52	e: 0.24 P:0.27	e: 0.044 P:0.84	e: −0.16 P:0.45	e: −0.08 P:0.70	e: −0.09 P:0.68
M2	e: 0.21 P:0.33	e: 0.08 P:0.08	e: 0.24 P:0.26	e: 0.18 P:0.40	e: −0.08 P:0.71	e: −0.11 P:0.63
M3	*e:* 0.083 *P:*0.71	e: 0.46 P:0.03∗	e: 0.26 P:0.23	e: −0.02 P:0.94	e: −0.12 P:0.59	e: −0.11 P:0.63
M4	e: 0.07 P: 0.74	e: 0.25 P:0.25	e: 0.24 P:0.25	e: 0.15 P:0.48	e: −0.13 P:0.54	e: 0.02 P:0.91
M5	e: 0.11 P:0.63	e: 0.28 P:0.20	e: 0.26 P:0.22	e: −0.16 P:0.44	e: −0.23 P:0.29	e: −0.10 P:0.65
Autoantibodies (Units/mL)	***HAD.D***	***S.Sum***	***S.PEM***	***BDI***	***MFS***	***FFS***
M1	e: ​−0.18 P:0.40	e: −0.01 P: 0.96	e: 0.03 P:0.89	e: ND P: ND	e: ND P: ND	e: ND P: ND
M2	e: −0.13 P:0.54	e: −0,03 P:0.91	e: 0.19 P:0.39	e: ND P: ND	e: ND P: ND	e: ND P: ND
M3	e: −0.14 P:0.52	e: 0.07 P:0.75	e: −0.08 P:0.72	e:0.53 P:0.06	e:0.03 P: 0.91	e: −0.17 P: 0.47
M4	e: −0.10 P:0.64	e:0.19 P: 0.37	e: 0.14 P:0.53	e:0.53 P:0.06	e:0.029 P: 0.91	e: −0.17 P: 0.47
M5	e: −0.1 P:0.39	e: 0.67 P:0.75	e: 0.02 P:0.92	e: ND P: ND	e: ND P: ND	e: ND P: ND

ND-not determined.

#### 3.2.1 Depression rate

HADS and Beck’s Depression Inventory (BDI) was used to measure patient depression rate. HADS was used for the SK -group and BDI for the Gottfries group. As shown by Table 2, patient mean scores for both anxiety and depression on HADS were lower than 7 (Mean: 4.91, SD:3.69), indicating a general absence of depression. Beck’s Depression Inventory (BDI) measured depression among the Gottfries patients and a mean score of 16.46 (SD:3.58) proposed the presence of mild depression.

#### 3.2.2 Symptomatology

The ME/CFS symptom related questionnaire (ME/CFS-SRQ) and the Fibro Fatigue Scale FFS) are designed to evaluate presence and severity of several ME/CFS- specific symptoms and were therefore used to investigate symptomatology and symptom severity within the two patient cohorts. No manuals, cut off values nor normative data exist for these two formulas and interpretation was based on clinical expertise from the treating physicians. Highest possible score on the ME/CFS symptom related questionnaire is 76 and the patient mean of 30.5 (SD: 9.91) is considered normal for the patient group. For the PEM-questions highest possible score is 8 and high values are expected. On the Fibro Fatigue Scale the highest possible score is 72 and the patient mean of 33.8 (SD:7.24) indicated a moderate symptomatology.

#### 3.2.3 Health related quality of life

Health Related Quality of Life was measured using RAND-36 and EQ-5D visual analogue scale (VAS). Patient values and population norms are presented in Table 4. Patient means were substantially lower than the norm for the physical summa index (RAND.PCS), physical functioning of RAND (RAND.PF) and for EQ-5D VAS. These results indicate a high degree of physical limitations and a low self-experienced quality of life among the SK-patients. Information about the health related quality of life among the Gottfries patients were not obtained evaluated by the collected questionnaires.

#### 3.2.4 Presence of fatigue

Fatigue was measured using Fatigue Severity Scale (FSS) and Mental Fatigue Scale (MFS). The FSS scores were compared to population norm value of 2.3 and as shown in Table 3 mean of patients were substantially higher, implying a state of severe fatigue. Analogous results were found for the mental fatigue scale with patient mean heavily outrunning the cut off value of 10.5.

### 3.3 Group characteristics and subgroups

Principal component analysis of antibody levels showed distinct differentiations between both patient cohorts and compared to controls as illustrated by PCA plots in Fig. 4 a-c. SK-patients were more similar to controls but still showed a pattern of scattered values with some extreme outliers while GC-patients differed significantly from both SK-patients and the controls. Based on the PCA plot in Fig. 4a it was possible to identify 2 subgroups within the SK-patients. The distinction between the two groups was made by using the 95% confidence interval for controls, visualized by an eclipse in the plot. Patients with scores outside of the confidence interval were defined as having divergent antibody levels compared to the patients laying inside of the interval, showed values more similar to the controls. 11 patients were identified as divergent and were grouped into group 1 while the rest of the 13 patients were defined as group 2.

Group 1 and 2 were compared regarding patient characteristics and questionnaire scores with the aim of identifying possible characteristics prone to cause the antibody level deviations, such as, co-morbidities and disease trigger, and to investigate whether deviant antibody levels seemed to be linked to a specific symptomatology or disease severity.

Mean values of age, gender, disease trigger and disease duration prior to sampling were similar within the 2 groups but the prevalence of co-morbidity equalled 73% in group 1 compared to 31% in group 2, which was a notable difference. However, the same two co-morbidities were the most frequent in both groups; Hypothyroidism and Fibromyalgia.

Mean value comparisons of patient questionnaire scores between group 1 and 2 revealed no distinct divergences, results are presented in Table 6.

**Table 6:** Mean and Standard Deviation (SD) on patient questionnaires for SK- subgroup 1 and 2. Results show mean value differences within one standard deviations for both groups, indicating absence of significant differences.

Groups	RAND.PCS	RAND.MCS	FSS	EQ.VAS	HAD.A	HAD.D	ME/CFS-SRQ
***Group*** 1 (n ​= ​11)	22.15 (SD:16)	45.49 (SD:12.19)	6.63 (SD:0.42)	31.18 (SD:13.14)	5.27 (SD:3.98)	7.18 (SD:3.60)	29.36 (SD:8.44)
***Group 2****(n* ​= ​*13)*	22.17 (SD:9.29)	44.19 (SD:9.31)	6.61 (SD:0.36)	32.34 (SD:17.41)	4.58 (SD:3.55)	7.33 (SD:3.73)	32.08 (SD:10.15)

## 4 Discussion

Our results revealed significantly increased levels of antibodies to M3 and M4 receptors in both patient cohorts and of antibodies to β1, β2, M3 and M4 receptors in one cohort. These results validate the results from Scheibenbogen et al. As a complement to prior research we found significantly increased levels of β1-autoantibodies in patients compared to controls and no detectable titres of muscarinic or adrenergic autoantibodies in cerebrospinal fluid, thus finding no evidence of intrathecal antibody production in the ME patient group.

Due to the heterogeneity of the ME-patient group few clinical findings of abnormal immunological markers have been uniformly shown and thus far it does not exist a ME-specific biomarker profile. The issue of patient heterogeneity in the aspect of creating a ME specific biomarker profile was recently highlighted in the EUROMENE Biomarker Landscape Project, a project designed to review and gather results from various clinical studies and investigate several potential biomarkers. A limited number of potential immunological markers were found and most of the reviewed studies showed dissonant results, lacked age and gender-matched controls or validation cohorts and had low evidence levels (Scheibenbogen et al., 2017).

Based on the definition of increased autoantibody levels as values above the 90th percentile of controls, Scheibenbogen et al. found increased levels of at least one of the autoantibody types in 29.5% of their patients. Using the same definition, we found that 91% of the Gottfries patients, 79% of the Stora Skondal patients and 25% of our controls had increased autoantibody levels. This provide evidence that there exists a general pattern of increased antibody levels to adrenergic and muscarinic receptors within the ME patient group. Taken together, the results show a characteristic deviation in plasma concentration of β1, β2, M3 and M4-receptor autoantibodies in a subgroup of the ME-patients compared to controls and a pattern of increased autoantibody levels to at least one of the adrenergic or muscarinic receptors within a great proportion of the patients.

Based on the normal function of adrenergic and muscarinic receptors and the role of autoantibodies directed towards neurotransmitter receptors in other autoimmune diseases with similar symptomatology as ME, such as POTS, Myasthenia Gravis and hypothyroidism, it has been considered possible that increased autoantibody levels might have clinical significance through interfering with the normal receptor function. The receptor-autoantibody interactions were expected to initiate immune system activation through B-cell stimulation, diminished autonomic nervous system activity and affection of metabolism through decreased lipolysis, gluconeogenesis and insulin secretion. In addition, binding of autoantibodies to muscarinic receptors is believed to interfere with acetylcholine release, thus impacting important CNS functions (Loebel et al., 2016).

Among the first evidence strengthening the theory about a pathological significance of autoantibodies in ME came from a clinical phase 2 study in Norway (Fluge et al., 2015). In this study Fluge et al. showed that CD20^+^ B-cell depletion by usage of the monoclonal anti- CD-20 antibody Rituximab had the potential of a partial or complete clinical remission in 62% of the patients. Responders experienced a 23 week delayed response with regression of fatigue, mental and physical limitations as well as a higher self-reported function. The 23 week delay suggested that the effects were mediated by depletion of short-lived antibody-producing plasma cells arising from CD20^+^ memory B-cells, resulting in a wash out of autoantibodies and argues towards an autoimmune pathogenesis and clinical significance of autoantibodies within a subgroup of ME-patients. Despite these promising results Fluge et al. were unable to replicate the results in their recently completed phase III study RituxME and yet again the complexity of patient heterogeneity was observed. The lack of consistent results challenges the appreciation of pathological significance of autoantibodies and the potential disease remission upon depletion and reveals an important knowledge gap (Fluge and Gurvin Rekeland, 2018).

This obvious knowledge gap motivated our attempts to find correlations between autoantibody levels and symptomatology/disease severity. Unlike prior studies we used a larger amount of questionnaires and generated a wider range of disease specific scores, thus investigating more aspects of patient’s disease profiles. The symptomatology in our two cohorts showed a typical ME-disease profile with low grades of depression and anxiety but severe fatigue, post exertional malaise, physical limitations and a very low subjective quality of life. However, no strong correlations between high autoantibody levels and disease severity were found. Our correlation analyses found no significant correlations between antibody levels and any of the symptom scores, suggesting that associations between levels of adrenergic and muscarinic receptor-antibodies and symptomatology are complex or non-existing.

The results of the PCA-analyses helped us to investigate associations between autoantibody level-patterns and disease severity, and did therefore complement the results from the correlation analyses. By observation of the different PCA-plots a couple of interesting results were found.

Firstly it became evident that the SK- and GC-patients were distinctively separated regarding autoantibody deviations, with more substantial deviations in M3 and M4 autoantibody levels within the SK-group and more substantial deviations of β1 and β2 autoantibody levels within the GC-patients (shown in Fig. 4c). In order to find any possible explanations behind the distinct differentiations in autoantibody deviations between the GC- and SK-patients we compared patient group characteristics and questionnaire scores but no obvious differences were found and it was therefore hard to draw any conclusions about either the reason behind the differences or their correlation to disease severity. Gender distribution was equal in both groups with 16 female patients and 8 male patients and the age distribution were very similar with a mean age of 40 (SD: 11) in the SK-group and 43 (SD:12.2) in the GC group. The prevalence of co-morbidities differed a little between the groups, 50% versus 33% but the same 2 co-morbidities; Hypothyroidism and Fibromyalgia were most frequent. The questionnaire scores on the two questionnaires that examined the symptom severity of different ME-specific symptoms; ME/CFS-SRQ and FFS were not directly comparable but mean scores of 30.5 (SD:9.91) on ME/CFS-SRQ and 33.8 (SD:7.24) on FFS indicated moderate symptom severity in both groups.

Secondly the results revealed two distinct subgroups within the SK-patient group, one showing distinct deviations in autoantibody levels compared to controls and the other showing autoantibody levels more similar to the controls. The questionnaires mean scores of both subgroups were very similar and did therefore not indicate any obvious connection between deviant antibody patterns and disease severity. The attempts to identify possible explanations behind the deviant antibody levels in group 1, other than ME itself, such as gender, age, co-morbidities, disease duration and disease trigger revealed only one disparity between our two subgroups. The rate of co-morbidity equalled 73% in group 1 compared to 31% in the group without deviant levels, suggesting that there where a subgroup of SK-patients with a higher degree of autoimmunity and there is a possibility that deviant antibody levels are partly caused by common ME related co-morbidities rather than ME itself.

This lack of clear correlation between antibody levels and clinical symptoms challenge the clinical relevance of autoantibodies to adrenergic and muscarinic receptors and their connection to the pathology of ME. However, it is possible that increased levels of muscarinic and adrenergic autoantibodies above a certain cut off, for instance the normal levels for controls, do contribute to symptomatology but that symptoms and autoantibody-amounts show a non-linear relationship above this cut off.

If this is the case patients may benefit from treatment diminishing or removing antibodies from plasma, something that have been suggested by a recent trial, performed by Scheibenbogen et al. In this trial a subset of severely ill ME patients with infection triggered disease and increased β2-receptor antibody levels were treated with Immunoadsorbtion (IA) directed against the β2 autoantibodies. The study yielded somewhat ambiguous but promising results. Disease specific scores were used as outcome measure and results showed a rapid improvement of several symptoms in 7/10 patients and a long term improvement, lasting for 6–12+ months in 3 of these patients. However, some of the patients experienced a gradual worsening of some of the symptoms towards the end of the treatment period and it is therefore quite to hard determine the potential of the IA as a treatment option (Scheibenbogen et al., 2018).

As an alternative to immunoadsorption, plasmapheresis/plasma exchange (PE) is a less selective process that removes not only pathogenic autoantibodies, cytokines and complements but other plasma components such as fibrinogen (Scheider-Gold et al., 2016).

Based on the proof of existence of a wide range of antibodies and cytokine abnormalities within ME it is possible that PE is an alternative to IA and that the technique has the potential as a treatment option, removing not only specific types of autoantibodies but a wider range of plasma proteins, prone to cause symptomatology and disease severity in ME.

Immunostimulation, e.g., with Staphylococcal vaccine, theoretically could induce tolerance to autoepitopes involved in ME/CFS pathogenesis. It was earlier reported to be effective in ME/CFS and fibromyalgia patients in controlled studies (Gottfries et al., 2009). The vaccine is, however, withdrawn from the market.

There are several limitations of our current study that need to be addressed in future studies in order to understand the potential significance of increased autoantibodies in ME/CFS.

The most obvious shortfalls in our current study were the limited population sizes and the facts that we collected samples from two separate clinics at two separate occasions and therefore were unable to test both groups for all of the 10 receptor types. Another obstacle related to the use of separate clinics was the usage of different clinical routine symptom formulas, which limits direct comparisons of symptomatology and disease severity between the two clinics. Most patients in both cohorts would fit in the moderate severity category in for example the ICC-criteria – with very few patients having severe or very severe ME according to this definition. This of course impacts the possibilities of finding a correlation between antibody levels and severity.

A more thorough and standardized investigation of disease severity, symptom profiles, co-morbidities and current medication in a larger population of ME patients is needed in future studies. Further studies are also motivated to investigate and characterize the potential biological effects of these autoantibodies using in for example vitro assays and tissue binding studies.

The methodology used in this study has a cross-sectional approach. Using a longitudinal methodology would have the potential of finding correlations between individual fluctuations in plasma antibody levels and symptom severity. This would be important for the understanding of the clinical significance of antibodies in ME.

## 5 Conclusion

We found significant increases in plasma autoantibody levels in ME patients compared to controls for M3 and M4 -receptors in two Swedish patient cohorts and in β1, β2, M3 and M4-receptors in one of the cohorts. These findings confirm previous findings of increased adrenergic and muscarinic autoantibodies in a German cohort of ME patients. No significant correlations were found between autoantibody levels and disease severity. No evidence was found for intrathecal antibody production in ME, as no autoantibodies were detected in cerebrospinal fluid (CSF) samples.

These findings support previous findings that there exists a general pattern of increased antibody levels to adrenergic and muscarinic receptors within the ME patient group.

However, the role of increased adrenergic and muscarinic receptor autoantibodies in the pathogenesis of ME is still unclear and further research is needed to evaluate the clinical significance of these findings.

## Declaration of competing interest

None of the authors have presorted on any conflict of interest.
