# Supplementary material for: Autoantibodies to beta-adrenergic and muscarinic cholinergic receptors in Myalgic Encephalomyelitis (ME) patients – A validation study in plasma and cerebrospinal fluid from two Swedish cohorts
Source: Brain Behav Immun Health. 2020 Jul 18;7:100107. doi: 10.1016/j.bbih.2020.100107 (PMC8474431; doi:10.1016/j.bbih.2020.100107)
Supplement: Multimedia component 1 [file mmc1.docx]

**Supplemental Material Appendix**

**Health related quality of life questionnaires**

Health related quality of life was measured, using RAND-36 and EQ-5D that were collected for the SK patients. No formulas evaluating quality of life were accessible for the GC-patients.

***RAND-36 - Item Short- Form Health survey- Physical functioning***

The RAND-36 Short-Form Health Survey- physical functioning is designed to measure a general health related life quality. It consists of 36 questions assessing daily activities such as climbing stairs, lifting and walking. Questions are designed to evaluate functions within eight different dimensions; Physical functioning (PF), role limitations due to physical health (RP), role limitations due to emotional problems (RE), energy/fatigue (VT), emotional well- being (MH), social functioning (SF), pain (BP) and general health (GH). Each category contains a certain number of items and different answer options. A manual exists and can be used to calculate scores and transform every item to a linear scale ranging from 0-100, where 100 equals to highest possible score, indicating good health status (1).

In order to simplify statistical analysis, it is possible to calculate sum indexes using the RAND HSI method were the eight dimensions are compressed into two summa indexes RAND.PCS (RAND physical component) and RAND.MCS (RAND mental component). These two components have been proven to explain >85% of the variations within the eight dimensions. High PCS and MCS scores indicate a good mental and physical health.

In this study, both dimension scores and summa index scores, were calculated and relevant results are presented in the result section (2).

One difficulty using the summa indexes; MCS and PCS for RAND 36 in a Swedish population is that norm values, scoring templates and scoring- definitions of differences do not exist in Sweden. This made it hard to interpret patient scores and evaluate the physical and mental health statuses. This obstacle was solved by using Norweigan norm values on RAND.MCS and RAND.PCS. The decision to use Norweigan data was based on manual- information stating that on group level the differences of summa indexes are negligible, making it possible to compare Swedish and Norweigan data (3).

***EQ-5D (EuroQuol)****EQ-5D (EuroQuol) i*s a health related questionnaire designed to measure individual health related quality of life based on five health dimensions; mobility, hygiene, main activity, pain/disability and depression/worry. Every dimension is investigated using one item and the patient is asked to answer the statement based on level of agreement/ disagreement. The answers are then coded and a score of 1, 2 or 3 is obtained. 1 is equal to no problem, 2 is equal to some problem and 3 is equal to severe problem and after the completion of the formula, a 5- digit number combination is acquired. The number combination can then be used to evaluate the patient’s health related life quality or to calculate an index score that can be used in statistical analyses. Included in the instrument is also a vertical visual analogue scale (VAS- scale) where the patient is asked to appreciate the current health status on a scale ranging from 0-100 where 100 represents the patient’s best individual health state. Swedish normative data for the VAS scale is available and was used for interpretation purposes (4, 5, 6)

**Fatigue**

The level of fatigue among the patients were measured foremost using the Fatigue Severity Scale and the Mental Fatigue Scale.

***Fatigue Severity Scale (FSS)***

Fatigue Severity Scale is a one-dimensional questionnaire containing 9 separate questions designed to capture the impact and severity of fatigue. Participants are asked to rate the 9 items on a 7 point Likert scale where 1 is equal to strong disagreement and 7 is equal to strong agreement. The final score is an average of the total sum score that can vary from 1-7. Scores >3.2 indicates presence of fatigue and scores greter than 4 indicates severe fatigue (7).

***Mental Fatigue Scale (MFS)***

Mental Fatigue Scale is a self-assessment scale developed in Gothenburg with the aim to help the individual/ Health care professionals to gain insight into the effect from mental fatigue. The questionnaire investigates the patient’s current health status during the past month and is constructed of 15 items. The patient is asked to evaluate the accurancy of each statement using 0-3 where 0 equals no issues with the symptom and 3 is equal to severe issues regarding the symptom. A cutoff score at 10,5 is recommended and scores greater than this value indictes presence of fatigue (8).

**Depression**

Evaluation of Depression-and Anxiety rate within the 2 patient groups was done using the Hospital Anxiety and Depression Scale and Beck’s Depression Inventory.

***Hospital Anxiety and Depression Scale (HADS)***

Hospital Anxiety and Depression Scale was developed to detect presence and severity of depression and/or anxiety in non-psychiatric settings. The questionnaire is composed of 14 questions were 7, (question; 1,3,5,7,9,11,13) are designed to evaluate anxiety and 7 (question 1,2,4,6,8,10,12,14) are designed to evaluate depression. Each question has four reply alternatives and the patient is asked to answer based on symptom-experiences during the previous two weeks. The answers are coded and a total score for each category; anxiety and depression is calculated. Clear cut-off values are available and should be used for interpretation. 0-7 points equals no depression or anxiety, 8-10 points suggests a possible state of depression/ anxiety, scores above or equal to 11 indicate a presumable state of anxiety/ depression and a score of 15-21 represents severe anxiety and/or depression (9).

***Beck’s Depression Inventory***

Becks Depression Inventory is designed to measure state of depression within psychiatric patients as well as incidence of depression within a population. The inventory consists of self-assessment questionnarie with 21 items designed to measure 21 separate symptoms and attitudes including; mood, pessimism, sense of failure, decreased satisfaction, guilt, punishment, self- contempt, suicidal thoughts, fret, social withdrawn, indecision, changed body image, working difficulties, insomnia, fatigue, decreased apetite, weight loss, anxiety, sexual disturbances Each item is graded 0-3 and a summa score is calculated. A score of 0-13 points indicates minimal depression, 14-19 indicates mild depression, 20-28 is valuated as moderate depression and a score of 26-63 is considered severe depression (10).

**Symptomatology and disease profile**

To gain comprehension about the patient’s disease profile, most prominent and most severe symptoms the ME/CFS Symptom Related Questionnaire (ME/CFS-SRQ) were collected for the SK-patients. Similar information for the GC- patients was obtained through the Fibro Fatigue Scale.

***ME/CFS Symptom Related Questionnaire (ME/CFS-SRQ)***

The Symptom Related Questionnaire for ME/CFS (ME/CFS-SRQ) is a Swedish questionnaire investigating severity of common symptoms that are part of the Canadian consensus criteria for ME/CFS; general pain, joint pain, morning rigidity, headache, fatigue, dizziness, tenderness, colon irritable, restless legs, bladder dysfunction, decreased memory, koncentration difficulties, sleep disturbances, chills/perspitations, paraesthesias, palpitations, feverishness, recurrent throat pain. The questionnaire consists of 21 items, each containing a Likert scale 1-4 where 1 is equal to no symptom and 4 represents severe symptom. 2 separate scores are calculated where questions 1-19 yield a summa score and question 21-22 are handeled separately and yields a score representing the occurence and severity of post exertional malaise (PEM). There exist no manual, cut off values or normative data for the ME/CFS symptom related questionnaire (ME/CFS-SRQ). Questionnaire instructions and score interpretations are based on clinical expertise. The questionnaire was primarily used to compare SK-patients with and without deviating autoantibodies. (11)

***Fibro Fatigue Scale (FFS)***

The Fibro Fatigue Scale is constructed to create an observer’s rating scale sensitive to change for measuring severity and treatment outcome in patients suffering from ME/CFS and fibromyalgia. The scale consists of 12 items that evaluate pain, fatigue, muscular tension, impaired memory, concentration difficulties, irritability, sleep disturbances, sadness autonomic dysfunction, irritable bowel syndrome, experience of infection and headache. Symptoms are rated on a scale from 0-6 where 0 equals to no symptoms and 6 is equvialent to severe symptoms. To facilitate scoring anchoring points are given for scores 0,2,4,6 but it is also possible to score in between these points thus yielding a score of 1,3,5. Grading is done through semi-structured interviews. There exist no manual cut-off values nor any normative data, which makes clinical expertise crucial for interpretation (12). Through personal contact with experienced clinicians at the Gottfries clinic, interpretation guidelines were given. According to these a score of 0-12 indicate no symptoms, 13-24 should be interpretated as mild symptoms, 25-36 equal moderate symptoms, 37-48 is equal to severe symptoms, 49-60 indicate very severe symptoms and a score greater than 61 implies that the person is bedridden.

**Quantification of patient’s questionnaires**

For each of the questionnaires, patient scores were calculated using manuals. This was done in order to deepen the understanding of disease profiles within our 2 cohorts. It was not possible to make direct comparisons of questionnaire scores from formulas selected at each clinic as they did not use the same formulas but they made it possible to get a blatant understanding of the symptom severity in each of the groups and associate it with antibody patterns. It would however have been preferable if the two clinics would have used identical symptom formulas as it would have enabled more precise comparisons.

For some of the questionnaires interpretation guidelines were explicitly described within the manuals and clear cut-off values were given but for formulas missing cut-off values and interpretation guidelines Swedish and Norweigan population norm values were used for interpretation purposes. Norm values were used for RAND-36, EQ-5D and FSS and all comparisons are presented in the result section. Calculation of individual questionnaire scores were done for all of the patients 1-48 in order to enable correlation analyses but are not induvidyally presented. Instead patient mean and standard deviation for the different formulas are presented in Table 3 and 4 in the main paper in order to provide a general clinical understanding of the two patient groups. The purpose of the correlation analyses between receptor autoantibody levels and questionnaire scores to explore possible correlations between high antibody and disease severity, something that could increase the understanding about clinical significance of the presence of autoantibodies, which in turn is important for deepening comprehension about the pathological mechanisms in ME/CFS and the potential of different treatment options.

Correlation analysis between the individual autoantibodies and the various questionnares are presented in Table 5 in the main paper.

**References Supplemental Material Appendix**

1. Hays, R. D. M., L.S. (2001). The RAND-36 Measure of Health-Related Quality of Life. *Annals of Medicine, 33*(5), 350-357. doi:10.3109/07853890109002089
2. Ware, J. E. K., M. Keller, SD. (1994). *SF-36 Physical and Mental Health Summary Scales: A User’s manual.* (Vol. 5). Boston, MA: Health Assessment Lab.
3. Garratt, A. M. S., K. . (2017). Measurement properties and normative data for the Norweigan SF-36: results from a general population survey. *Health and Quality of Life Outcomes, 15*(1), 1-10. doi:0.1186/s12955-017-0625-9.
4. Registercentrum Sydost. (2017). *Instrument för att mäta generell hälsorelaterad livskvalitet (HRQoL).* Retrived from: <http://rcso.se/wp-content/uploads/2015/10/promcenter_sammanfattning_avseende_eq-5d_och_sf-36.pdf>. Visited 20191226.
5. Uppdrag Psykisk Hälsa. (1995).*EQ-5D.* Retrived from: <https://www.uppdragpsykiskhalsa.se/assets/uploads/2018/02/EQ-5D.pdf>. Visited 20191226.
6. Szende, A. J., B. Cabasés, J.(2014). *Self-Reported Population Health: An International Perspective based on EQ-5D.* Retrived from: <https://eq-5dpublications.euroqol.org/download?id=0_54006&fileId=54415>. Visited 20191226.
7. Krupp, B.L. *Fatigue Severity Scale (FSS).* Retrived from: <http://nesportandspine.com/sites/default/files/fss.pdf>. Visited 20191226.
8. University of Gothenburg, Sundén, P.(2015).Mental Fatigue Assessment, Subjective & objective methods. Retrived from: <https://mf.gu.se/english/assessment>. Visited 20191226.
9. Snaith, R. P., & Zigmond, A. S. (1986). The hospital anxiety and depression scale. *British medical journal (Clinical research ed.)*, *292*(6516), 344.
10. Socialstyrelsen.(2018)*.* Becks depression inventory. Retrived from:

<http://www.socialstyrelsen.se/evidensbaseradpraktik/sokimetodguidenforsocialtarbete/bdi>. Visited 20191226.

1. Martin A. Jonsjö, Rikard K. Wicksell, Linda Holmström, Anna Andreasson, Indre Bileviciute-Ljungar & Gunnar L. Olsson (2017) Identifying symptom subgroups in patients with ME/CFS – relationships to functioning and quality of life, Fatigue: Biomedicine, Health & Behavior, 5:1, 33-42, DOI: 10.1080/21641846.2017.1287546
2. Zachrisson, O. R., B. Jahreskog, M. Kron, Margareta. Gottfries C.G. (2002). A rating scale for fibromyalgia and chronic fatigue syndrome (the FibroFatigue scale). *Journal of Psychosomatic Research, 52*(6), 501-509. doi:10.1016/S0022-3999(01)00315-4
